# Low Albumin, Low Bilirubin, and High Alfa-Fetoprotein Are Associated with a Rapid Renal Function Decline in a Large Population Follow-Up Study

**DOI:** 10.3390/jpm11080781

**Published:** 2021-08-11

**Authors:** Wei-Yu Su, Neng-Sheng Chu, Jiun-Chi Huang, Pei-Yu Wu, Wen-Hsien Lee, Yi-Hsueh Liu, Szu-Chia Chen, Ho-Ming Su

**Affiliations:** 1 Department of Internal Medicine, Kaohsiung Medical University Hospital, Kaohsiung Medical University, Kaohsiung 807, Taiwan; s952135@gmail.com; 2 Department of Internal Medicine, Kaohsiung Municipal Siaogang Hospital, Kaohsiung 812, Taiwan; nengsheng@yahoo.com.tw (N.-S.C.); karajan77@gmail.com (J.-C.H.); wpuw17@gmail.com (P.-Y.W.); cooky-kmu@yahoo.com.tw (W.-H.L.); Liuboy17@gmail.com (Y.-H.L.); 3 Division of Gastroenterology, Department of Internal Medicine, Kaohsiung Medical University Hospital, Kaohsiung Medical University, Kaohsiung 807, Taiwan; 4 Division of Nephrology, Department of Internal Medicine, Kaohsiung Medical University Hospital, Kaohsiung Medical University, Kaohsiung 807, Taiwan; 5 Faculty of Medicine, College of Medicine, Kaohsiung Medical University, Kaohsiung 807, Taiwan; 6 Division of Cardiology, Department of Internal Medicine, Kaohsiung Medical University Hospital, Kaohsiung Medical University, Kaohsiung 807, Taiwan; 7 Research Center for Environmental Medicine, Kaohsiung Medical University, Kaohsiung 807, Taiwan

**Keywords:** rapid renal decline, liver function, follow-up

## Abstract

A rapid decline in renal function is associated with high cardiovascular morbidity and mortality, and therefore it is important to identify those at high-risk of rapid renal function decline. The relationship between liver function and renal function is unclear. Therefore, in this longitudinal study, we aimed to investigate associations between liver function and rapid renal function decline. A total of 27,116 participants were enrolled from the Taiwan Biobank and followed for 3.8 years. A rapid decline in renal function was defined as a decline in estimated glomerular filtration rate (eGFR) of ≥25%. Binary logistic regression analysis was used to identify associations between liver function parameters (glutamic-oxalacetic transaminase, glutamic-pyruvic transaminase, albumin, α-fetoprotein [AFP], total bilirubin, and gamma-glutamyl transpeptidase) and eGFR decline ≥ 25%. The rate of eGFR decline of ≥25% was 4.7%. Multivariable analysis showed that low albumin (odds ratio [OR], 0.173; *p* < 0.001), high AFP (OR, 1.006; *p* = 0.010), and low total bilirubin (OR, 0.588; *p* < 0.001) were significantly associated with eGFR decline ≥ 25% in all study participants. After excluding abnormal liver function, low albumin (OR, 0.189; *p* < 0.001), high AFP (OR, 1.007; *p* = 0.011), and low total bilirubin (OR, 0.569; *p* = 0.001) were still significantly associated with an eGFR decline of ≥25%. The results of this large population-based cohort study showed associations between low albumin, low bilirubin, and high AFP with a rapid renal function decline. A greater understanding of potential risk factors for a rapid decline in renal function may help to reduce the burden of renal failure in this high-risk population.

## 1. Introduction

The incidence and prevalence of end-stage renal disease (ESRD) in Taiwan continue to increase [1], and significantly higher rates of cardiovascular morbidity and mortality have been reported in patients with ESRD compared to the general population [2]. In addition, a rapid decline in renal function has also been significantly associated with high cardiovascular morbidity and mortality, independent of the renal function at baseline [3,4]. Accordingly, it is vital to identify those potentially at high risk of a rapid decline in renal function so that optimal treatment can be provided.

Previous studies have reported that factors associated with renal function decline include old age, male sex, high systolic blood pressure, low diastolic blood pressure, hyperuricemia, proteinuria, hypoalbuminemia, diabetes mellitus, anemia, dyslipidemia, and cardiovascular disease [5,6,7]. Recent studies have shed light on the relationship between liver function and renal function decline. Patients with advanced liver diseases, cirrhosis, and failure have been associated with functional renal impairment, a condition known as hepatorenal syndrome (HRS). The pathophysiology of HRS involves vasoconstriction of the renal circulation causing splanchnic vasodilatation, portal hypertension, and activation of the sympathetic nervous and renin-angiotensin-aldosterone systems [8]. In a previous cohort study, Gines et al. reported that only hyponatremia, high plasma renin activity, and absence of hepatomegaly were independent significant predictors of HRS in patients with liver cirrhosis and ascites [9]. In addition, liver function parameters, including glutamic-oxalacetic transaminase (GOT), glutamic-pyruvic transaminase (GPT), gamma-glutamyl transpeptidase (γ-GT), bilirubin, albumin, prothrombin time, and Child–Pugh score were not found to have predictive value in their study [9]. In contrast, Janicko et al. reported that a higher bilirubin level was a significant and independent predictor of HRS in patients with alcoholic liver cirrhosis [10]. In addition, a post-hoc analysis of the reduction in endpoints in non-insulin-dependent diabetes mellitus with the Angiotensin II Antagonist Losartan trial and the Irbesartan Diabetic Nephropathy Trial both reported associations between a high bilirubin level and decreased composite renal endpoint of a two-fold increase in serum creatinine or ESRD among patients with diabetic nephropathy. This supports the emerging evidence that high bilirubin may protect against a decline in renal function [11,12]. However, high bilirubin was associated with better renal outcomes but faster annual decline in renal function in an Australian study [13].

Tumor markers are widely used to assist in making a diagnosis and monitoring certain tumors. Tumors can cause abnormal renal function [14,15,16], and chronic kidney disease (CKD) may increase the incidence of malignancy [16]. A previous study found no associations between serum carcinoembryonic antigen (CEA), cancer antigen (CA) 125, mucin 1, cell surface-associated protein, α-fetoprotein (AFP), neuron-specific enolase (NSE), and CA19-9 with serum creatinine in healthy individuals [17]. An earlier study also reported that CKD and hemodialysis did not affect the serum levels of AFP [18]. In addition, Xiaofang et al. demonstrated that, in CKD patients without benign or malignant tumors, the serum concentrations of CA19-9, CA125 (in males), cytokeratin fragment 21-1, NSE, and squamous cell carcinoma antigen were positively correlated with renal function decline, while there were no significant differences in the concentrations of CA125 (in females), AFP, CA15.3 or CA72.4 [19].

Large cohort follow-up studies on the associations between liver function parameters and renal outcomes are lacking. Therefore, due to the inconsistent results of previous studies and the lack of large cohort follow-up studies, the aim of this longitudinal study was to investigate associations among liver function tests and AFP with a rapid renal function decline defined as an estimated glomerular filtration rate (eGFR) decline of ≥25%.

## 2. Methods

### 2.1. Data Source and Collection

Data for this study were obtained from the Taiwan Biobank (TWB), which is the largest biobank in Taiwan. The TWB was established by the Taiwanese government to record lifestyle and genomic data of community-based Taiwanese residents aged 30 to 70 years with no history of cancer [20,21].

All of the participants registered in the TWB were interviewed by a TWB researcher to obtain information on age, sex, personal, and family medical histories including diabetes and hypertension, lifestyle factors, and diet. The participants also underwent physical examinations, during which data on body height, body weight, body mass index (BMI), systolic and diastolic blood pressures, and resting heart rate were recorded. Overnight fasting blood samples were also obtained from each participant, and data on fasting glucose, total cholesterol, triglycerides, uric acid, serum creatinine, hemoglobin, GOT, GPT, albumin, AFP, total bilirubin, γ-GT, hepatitis B surface antigen, and anti-hepatitis C virus antibody were recorded.

### 2.2. Study Patients

A total of 104,451 participants were enrolled in the TWB between 2012 and 2018. In this study, we only included those with complete data at baseline and after 3.8 years of the follow-up (*n* = 27,116; 9599 men and 17,517 women).

### 2.3. Definition of Rapid Renal Function Decline

EGFR was calculated using the 4-variable Modification of Diet in Renal Disease study equation [22]. A rapid decline in renal function was defined as a decline in eGFR by ≥25% [23]. The percentage decline in eGFR was calculated as (baseline eGFR—follow-up eGFR)/baseline eGFR.

### 2.4. Ethics Statement

The Institutional Review Board (IRB) on Biomedical Science Research, Academia Sinica, Taiwan, and the Ethics and Governance Council of the TWB gave ethical approval for the TWB. In addition, all participants in the TWB signed informed consent forms. The current study was approved by the IRB of Kaohsiung Medical University Hospital (KMUHIRB-E(I)-20190398) and conducted following the principles of the Declaration of Helsinki.

### 2.5. Statistical Analysis

Categorical variables are expressed as percentages, and continuous variables are expressed as mean ± standard deviation. The study patients were classified into two groups according to an eGFR decline of ≥25% or <25%. The independent *t*-test was used to analyze differences in continuous variables between groups, and the chi-square test was used for categorical variables. Factors associated with an eGFR decline of ≥25% were identified using univariable and multivariable logistic regression analyses, with the statistically significant variables in univariable analysis being entered into the multivariable analysis. A *p* value of <0.05 was considered to be significant. All statistical analyses were performed using SPSS for Windows (version 26.0, SPSS Inc. Chicago, IL, USA).

## 3. Results

A total of 27,116 participants (9599 men and 17,517 women; mean age 51 ± 10 years) were included, including 1280 in the eGFR decline ≥ 25% group and 25,836 in the <25% group. The rate of eGFR decline ≥ 25% was 4.7% (1280/27,116).

### 3.1. Comparisons of Baseline Characteristics between the Participants with an eGFR Decline of ≥25% and <25%

Comparisons of baseline characteristics between the eGFR decline of ≥25% and <25% groups are shown in Table 1. Compared to the eGFR decline < 25% group, the eGFR decline ≥ 25% group were older, more predominantly female, and had higher prevalence rates of hypertension and diabetes mellitus, higher systolic and diastolic blood pressures, higher heart rate, higher BMI, higher levels of fasting glucose and triglycerides, lower levels of uric acid, total cholesterol and hemoglobin, higher baseline eGFR, and lower follow-up eGFR. With regards to the liver-related parameters, the eGFR decline ≥ 25% group had higher GOT, AFP and γ-GT, and lower albumin and total bilirubin.

### 3.2. Determinants Associated with an eGFR Decline of ≥25% in All Study Participants

Table 2 presents the univariable logistic analysis of the factors associated with an eGFR decline of ≥25% in all study participants. Old age, male sex, hypertension, diabetes mellitus, high systolic and diastolic blood pressures, high heart rate, high BMI, high baseline eGFR and high levels of fasting glucose, triglycerides, GOT, AFP and γ-GT, and low levels of uric acid, total cholesterol, hemoglobin, albumin, and total bilirubin were associated with an eGFR decline of ≥25%.

Table 3 presents the multivariable logistic analysis of the factors associated with an eGFR decline of ≥25% in all study participants. After adjusting for the significant variables in the univariable analysis (Table 2), old age, hypertension, diabetes mellitus, high systolic blood pressure, high fasting glucose, high uric acid, low hemoglobin, high baseline eGFR, low albumin (odds ratio [OR], 0.173; 95% confidence interval [CI], 0.127 to 0.236; *p* < 0.001), high AFP (OR, 1.006; 95% CI, 1.001 to 1.011; *p* = 0.010), and low total bilirubin (OR, 0.588; 95% CI, 0.439 to 0.786; *p* < 0.001) were significantly associated with an eGFR decline of ≥25%.

### 3.3. Determinants Associated with an eGFR Decline of ≥25% in the Male Participants with GPT ≤ 35 μ/L and Female Participants with GPT ≤ 25 μ/L

Table 4 shows the results of multivariable logistic analysis of the factors associated with an eGFR decline of ≥25% in the male participants with GPT of ≤35 μ/L and female participants with GPT of ≤25 μ/L (*n* = 21,715) (excluding those with an abnormal liver function [24]). After adjusting for the significant variables in the univariable analysis (Table 2), hypertension, diabetes mellitus, high systolic blood pressure, high heart rate, high fasting glucose, low hemoglobin, high baseline eGFR, low albumin (OR, 0.189; 95% CI, 0.130 to 0.274; *p* < 0.001), high AFP (OR, 1.007; 95% CI, 1.002 to 1.012; *p* = 0.011), and low total bilirubin (OR, 0.569; 95% CI, 0.405 to 0.801; *p* = 0.001) were significantly associated with an eGFR of decline ≥25% in the male participants with GPT of ≤35 μ/L and female participants with GPT of ≤25 μ/L.

Covariates in the multivariable model included age, gender, hypertension, diabetes mellitus, systolic and diastolic blood pressures, heart rate, body mass index, fasting glucose, uric acid, total cholesterol, triglyceride, hemoglobin, baseline eGFR, GOT, albumin, AFP, total bilirubin, and γ-GT (significant variables in the univariable analysis in Table 2).

## 4. Discussion

In this longitudinal analysis, we investigated the associations between liver function parameters and renal function decline among 27,116 participants who were followed for a median of 3.8 years in the TWB. Overall, we found that, after adjusting for confounders, the participants with low albumin, low bilirubin, and high AFP were associated with a rapid decline in renal function (eGFR decline ≥ 25%).

There are several important findings to this study. First, a low albumin level was associated with a rapid renal function decline. Several studies have reported associations between lower serum albumin concentrations and kidney function decline, risk of incident CKD, and progression to ESRD [13,25,26,27]. Potential mechanisms for hypoalbuminemia include decreased supply of amino acids, impaired liver synthesis, increased renal loss, and increased tissue catabolism or distributional problems [28]. The molecular mechanisms underlying a decrease in albumin synthesis in the liver have been attributed to the effects of cytokines such as interleukin (IL)-1 [29], IL-6, and tumor necrosis factor (TNF)-*α* [30]. Albumin synthesis in hepatocyte requires mRNA for translation [30]. The mRNA concentration available for action on ribosomes is an important factor controlling the rate of albumin synthesis. Cytokines decrease the gene transcription, leading to a reduction in albumin mRNA concentration [30]. IL-6 is known to play a role in different etiologies of renal disease, and the increased generation of IL-6 due to oxidative stress, chronic inflammation and fluid overload, and reduced clearance of IL-6 due to impaired renal function have been shown to contribute to elevated plasma IL-6 levels in CKD patients [31]. IL-6 causes endothelial injury mainly by reducing the expressions of endothelial nitric oxide synthase and adiponectin (an anti-atherogenic adipokine), thereby contributing to an increased incidence of chronic vascular disease, progression of CKD, and a rapid renal function decline [27,31]. In addition, TNF-α was associated with a rapid renal function decline in the Chronic Renal Insufficiency Cohort Study [27]. Inflammatory cytokines, including TNF-α, IL-1, IL-6, and IL-18, are involved in signaling cascades which can lead to a vicious circle of inflammation-associated injuries in the tubulointerstitial and glomerular compartments of the kidney [32]. In addition, TNF-α can increase inflammatory responses in the kidneys by elevating the expressions of adhesion molecules and inducing the production of growth factors, other cytokines, and proinflammatory chemokines such as macrophage colony-stimulating factor, monocyte chemoattractant protein-1 and IL-8 in an autocrine and paracrine manner [32]. Therefore, in addition to renal loss due to kidney injury resulting in low serum albumin [33], inflammatory cytokines also play crucial roles in both albumin synthesis and renal function progression.

Second, low bilirubin was associated with a rapid renal function decline. A prospective observational study reported an association between low bilirubin concentration and the composite of a two-fold increase in serum creatinine or ESRD requiring dialysis in Japanese patients with stages 3–5 CKD [12]. Nevertheless, some studies have reported opposite findings [13,34,35]. Majoni et al. reported that a high bilirubin concentration was associated with a faster annual decline in eGFR but better renal outcomes in an Australian population [13]. A cross-sectional study conducted in the United States also demonstrated associations between a high bilirubin concentration and low eGFR and increased albuminuria in healthy adults [34]. In addition, a large retrospective study reported that hyperbilirubinemia (total bilirubin > 2.0 mg/dL) was an independent risk factor for contrast-induced acute kidney injury, dialysis, and mortality after receiving contrast-enhanced computed tomography [35].

The exact causal relationship between bilirubin and kidney function remains unclear. Several factors have been shown to affect bilirubin concentration, including cigarette smoking, sex, fasting, drugs, altitude, age, and race [36]. Bilirubin is a metabolic product of heme degradation by heme oxygenases (HOs), and especially HO-1. HOs convert heme to biliverdin, which is then reduced to bilirubin by biliverdin reductase [36]. HOs play an important role in reducing reactive oxygen species (ROS) production by producing bilirubin, degrading heme, and releasing free iron [36]. Moreover, bilirubin has been shown to inhibit NADPH oxidase activity in vitro [37]. Accordingly, these findings show that bilirubin is a potent antioxidant under physiological conditions. Oxidative stress is increased in kidney disease due to both antioxidant depletion and an increase in ROS production [38]. In addition, oxidative stress can accelerate the progression of kidney disease, thereby creating a vicious circle of CKD progression [38]. High oxidative stress leads to low bilirubin caused by oxidation into biliverdin [39] and low albumin caused by decrease synthesis by the liver [30]. Taken together, these findings indicate an association between a high bilirubin level and the attenuation of renal damage.

Third, high AFP was associated with a rapid renal function decline. Previous studies have not found associations between serum AFP and renal function in healthy individuals, as well as patients with CKD, ESRD, undergoing hemodialysis, peritoneal dialysis, and renal transplantation [17,18,19]. AFP is a protein produced by the fetal liver and yolk sac and subsequently mainly by the adult liver, with levels gradually decreasing after birth to below 15 ng/mL in normal adults [40]. The concentration of AFP has reportedly increased by up to 80% in patients with various types of cancers; however, an increase in serum AFP up to 400 ng/mL has also been reported in benign conditions such as liver cirrhosis, acute and chronic hepatitis, and pregnancy [41]. Chen et al. reported a higher prevalence of advanced CKD in patients with liver cirrhosis and higher model for end-stage liver disease scores [42]. Nationwide cohorts from Taiwan have reported associations between chronic hepatitis B virus infection and increased risks of CKD and ESRD [43,44]. Therefore, we hypothesize that the association between a high AFP level and a rapid renal function decline in individuals without cancer may, at least in part, be due to chronic liver disease.

Another interesting finding of this study is that a high heart rate was also associated with rapid renal function decline. Previous studies have reported associations between a high heart rate and hypertension, acute coronary syndrome, heart failure [45], and cardiovascular death [46]. Another study reported that a high heart rate could predict a higher risk of future microvascular complications, including both nephropathy and retinopathy in patients with type 2 diabetes mellitus [47]. Moreover, a post-hoc analysis by Böhm et al. showed associations between a high heart rate and poor renal outcomes, including microalbuminuria, macroalbuminuria, doubling of creatinine, and the development of ESRD in patients with cardiovascular disease, cerebrovascular disease, and high-risk diabetes with end-organ damage [48]. In addition to studies performed in untreated populations, Kolloch et al. analyzed patients treated with either heart rate-slowing calcium antagonists or β-blockers [45], and found that elderly hypertensive patients with stable coronary artery disease who received heart-rate-lowering treatment had a lower incidence of adverse outcomes [45]. Several potential mechanisms for the association between a high heart rate and a rapid renal function decline have been proposed. For example, the number of arterial pulse waves caused by a high heart rate may contribute to glomerular damage [49]. In addition, an increased heart rate has also been shown to affect endothelial function, leading to increased oxidative stress and resulting in atherosclerosis [50]. In addition to oxidative stress, the progression of atherosclerosis has also been associated with an acceleration in kidney disease progression [38,51]. The finding in the present study of an association between a high heart rate and an increased risk of a rapid renal function decline suggest that such patients should be followed up closely and possibly given more aggressive treatment.

The main strengths of this study are the large-scale investigation and follow-up of the associations between liver function parameters and a renal function decline. However, there are also several limitations to this study. Data on medication history including anti-diabetic, antihypertensive, and lipid-lowering agents were not available in the TWB, and they could also have affected longitudinal changes in renal function. Therefore, we cannot exclude the impact of such medications on our results. In addition, information on some factors, such as proteinuria, which may have contributed to a rapid renal function decline, was limited. Finally, according to statistics from the TWB, about 50% of the participants return for follow-up measurements, which may have resulted in sampling bias, and this may have affected the interpretation of our results.

## 5. Conclusions

In this large population-based cohort study, we identified associations between low albumin, low bilirubin, and high AFP with a rapid renal function decline. A greater understanding of the potential risk factors for a rapid renal function decline may help reduce the burden of renal failure in this high-risk population.

## Figures and Tables

**Table 1:** Comparison of baseline characteristics between participants with eGFR decline of ≥25% and <25%.

Characteristics	eGFR Decline ≥ 25% (*n* = 1280)	eGFR Decline < 25% (*n* = 25,836)	*p*	All (*n* = 27,116)
Age (year)	52 ± 11	51 ± 10	<0.001	51 ± 10
Male gender (%)	27.0	35.8	<0.001	35.4
Hypertension (%)	23.8	12.6	<0.001	13.1
Diabetes mellitus (%)	13.9	4.9	<0.001	5.3
Systolic blood pressure (mmHg)	122 ± 20	117 ± 18	<0.001	118 ± 18
Diastolic blood pressure (mmHg)	73 ± 11	72 ± 11	0.012	73 ± 11
Heart rate (beat/min)	71 ± 10	69 ± 9	<0.001	69 ± 9
Body mass index (kg/m^2^)	24.5 ± 4.0	24.1 ± 3.5	<0.001	24.1 ± 3.6
Fasting glucose (g/dL)	103 ± 35	96 ± 19	<0.001	96 ± 20
Uric acid (mg/dL)	5.3 ± 1.5	5.5 ± 1.4	<0.001	5.5 ± 1.4
Total cholesterol (mg/dL)	193 ± 37	196 ± 35	0.003	195 ± 35
Triglyceride (mg/dL)	122 ± 86	114 ± 83	0.001	114 ± 83
Hemoglobin (g/dL)	13.4 ± 1.5	13.8 ± 1.6	<0.001	13.7 ± 1.6
eGFR (mL/min/1.73 m^2^, baseline)	128.5 ± 40.3	108.3 ± 24.3	<0.001	109.2 ± 25.6
eGFR (mL/min/1.73 m^2^, follow-up)	86.7 ± 29.0	107.6 ± 25.3	<0.001	106.6 ± 25.8
Liver related parameters				
GOT (μ/L)	25.5 ± 15.6	24.7 ± 11.6	0.022	24.7 ± 11.8
GPT (μ/L)	24.0 ± 19.5	23.8 ± 19.4	0.645	23.8 ± 19.4
Albumin (g/dL)	4.43 ± 0.26	4.56 ± 0.23	<0.001	4.55 ± 0.23
AFP (ng/mL)	7.0 ± 29.0	3.6 ± 5.2	<0.001	3.79 ± 8.01
Total bilirubin (mg/dL)	0.61 ± 0.29	0.68 ± 0.28	<0.001	0.66 ± 0.28
γ-GT (μ/L)	27 ± 45	24 ± 28	0.003	24 ± 29
HBsAg positive (%)	10.1	11.8	0.080	11.7
Anti-HCV antibody positive (%)	2.8	2.9	0.948	2.8

Abbreviations. eGFR, estimated glomerular filtration rate; GOT, glutamic-oxalocetic transaminase; GPT, glutamic-pyruvic transaminase; AFP, α-fetoprotein; γ-GT, gamma-glutamyl transpeptidace; HBsAg, hepatitis B surface antigen; HCV, Hepatitis C virus.

**Table 2:** Parameters associated with an eGFR decline of ≥25% in univariable binary logistic analysis in all study participants (*n* = 27,116).

Parameters	Odds Ratio (95% CI)	*p*
Age (per 1 year)	1.010 (1.005–1.016)	<0.001
Male (vs. female)	0.664 (0.585–0.753)	<0.001
Hypertension	2.167 (1.895–2.477)	<0.001
Diabetes mellitus	3.145 (2.659–3.721)	<0.001
Systolic blood pressure (per 1 mmHg)	1.013 (1.010–1.016)	<0.001
Diastolic blood pressure (per 1 mmHg)	1.007 (1.001–1.012)	0.012
Heart rate (per 1 beat/min)	1.017 (1.011–1.023)	<0.001
Body mass index (per 1 kg/m^2^)	1.036 (1.021–1.052)	<0.001
Fasting glucose (per 1 g/dL)	1.010 (1.008–1.012)	<0.001
Uric acid (per 1 mg/dL)	0.890 (0.854–0.928)	<0.001
Total cholesterol (per 1 mg/dL)	0.998 (0.996–0.999)	0.003
Triglyceride (per 1 mg/dL)	1.001 (1.000–1.001)	0.001
Hemoglobin (per 1 g/dL)	0.853 (0.824–0.883)	<0.001
Baseline eGFR (per 1 mL/min/1.73 m^2^)	1.025 (1.023–1.027)	<0.001
Liver related parameters		
GOT (per 1 μ/L)	1.004 (1.001–1.008)	0.023
GPT (per 1 μ/L)	1.001 (0.998–1.003)	0.645
Albumin (per 1 g/dL)	0.102 (0.081–0.130)	<0.001
AFP (per 1 ng/mL)	1.018 (1.013–1.024)	<0.001
Total bilirubin (per 1 mg/dL)	0.412 (0.324–0.515)	<0.001
γ-GT (per 1 μ/L)	1.002 (1.001–1.003)	0.004
HBsAg positive	0.847 (0.702–1.020)	0.080
Anti-HCV antibody positive	0.989 (0.704–1.388)	0.948

Values expressed as odds ratio and 95% confidence interval (CI). Abbreviations are the same as in Table 1.

**Table 3:** Parameters associated with an eGFR decline of ≥25% in multivariate binary logistic analysis in all study participants (*n* = 27,116).

Parameters	Odds Ratio (95% CI)	*p*
Age (per 1 year)	1.009 (1.001–1.018)	0.035
Male (vs. female)	0.840 (0.687–1.027)	0.089
Hypertension	1.901 (1.578–2.290)	<0.001
Diabetes mellitus	1.965 (1.519–2.543)	<0.001
Systolic blood pressure (per 1 mmHg)	1.011 (1.005–1.017)	0.001
Diastolic blood pressure (per 1 mmHg)	1.000 (0.990–1.010)	0.930
Heart rate (per 1 beat/min)	1.005 (0.998–1.012)	0.166
Body mass index (per 1 kg/m^2^)	0.995 (0.975–1.016)	0.647
Fasting glucose (per 1 g/dL)	1.004 (1.002–1.007)	0.001
Uric acid (per 1 mg/dL)	1.075 (1.009–1.144)	0.025
Total cholesterol (per 1 mg/dL)	1.001 (0.999–1.003)	0.472
Triglyceride (per 1 mg/dL)	1.001 (1.000–1.001)	0.055
Hemoglobin (per 1 g/dL)	0.884 (0.836–0.935)	<0.001
Baseline eGFR (per 1 mL/min/1.73 m^2^)	1.025 (1.022–1.028)	<0.001
Liver related parameters		
GOT (per 1 μ/L)	1.002 (0.997–1.006)	0.524
GPT (per 1 μ/L)	-	-
Albumin (per 1 g/dL)	0.173 (0.127–0.236)	<0.001
AFP (per 1 ng/mL)	1.006 (1.001–1.011)	0.010
Total bilirubin (per 1 mg/dL)	0.588 (0.439–0.786)	<0.001
γ-GT (per 1 μ/L)	1.001 (0.999–1.003)	0.360
HBsAg positive	-	-
Anti-HCV antibody positive	-	-

Values expressed as odds ratio and 95% confidence interval (CI). Abbreviations are the same as in Table 1.

**Table 4:** Parameters associated with the eGFR decline of ≥25% in multivariable binary logistic analysis in study participants with a GPT of ≤35 μ/L in males and ≤25 μ/L in females (*n* = 21,715).

Parameters	Odds Ratio (95% CI)	*p*
Age (per 1 year)	1.009 (0.998–1.019)	0.100
Male (vs. female)	1.157 (0.915–1.463)	0.224
Hypertension	1.897 (1.518–2.372)	<0.001
Diabetes mellitus	1.745 (1.266–2.405)	0.001
Systolic blood pressure (per 1 mmHg)	1.010 (1.003–1.017)	0.006
Diastolic blood pressure (per 1 mmHg)	0.999 (0.987–1.010)	0.844
Heart rate (per 1 beat/min)	1.009 (1.000–1.017)	0.006
Body mass index (per 1 kg/m^2^)	0.996 (0.971–1.022)	0.770
Fasting glucose (per 1 g/dL)	1.006 (1.003–1.009)	<0.001
Uric acid (per 1 mg/dL)	1.075 (0.997–1.158)	0.059
Total cholesterol (per 1 mg/dL)	1.001 (0.998–1.003)	0.548
Triglyceride (per 1 mg/dL)	1.000 (0.999–1.001)	0.476
Hemoglobin (per 1 g/dL)	0.886 (0.832–0.943)	<0.001
Baseline eGFR (per 1 mL/min/1.73 m^2^)	1.025 (1.022–1.028)	<0.001
Liver related parameters		
GOT (per 1 μ/L)	1.005 (0.988–1.023)	0.574
GPT (per 1 μ/L)	-	-
Albumin (per 1 g/dL)	0.189 (0.130–0.274)	<0.001
AFP (per 1 ng/mL)	1.007 (1.002–1.012)	0.011
Total bilirubin (per 1 mg/dL)	0.569 (0.405–0.801)	0.001
γ-GT (per 1 μ/L)	1.004 (1.000–1.008)	0.076
HBsAg positive	-	-
Anti-HCV antibody positive	-	-

Values expressed as odds ratio and 95% confidence interval (CI). Abbreviations are the same as in Table 1.

## Data Availability

The data underlying this study is from the Taiwan Biobank. Due to restrictions placed on the data by the Personal Information Protection Act of Taiwan, the minimal data set cannot be made pub-licly available. Data may be available upon request to interested researchers. Please send data re-quests to: Szu-Chia Chen, PhD, MD. Division of Nephrology, Department of Internal Medicine, Kaohsiung Medical University Hospital, Kaohsiung Medical University.
